# 

**Published:** 1848-01

**Authors:** 


TO THE MEMBERS OF THE MISSISSIPPI VALLEY ASSOCIATION OF DENTAL SURGEONS.

For the information of members of the Society, who were not present at the last annual meeting, we would state that the fact of being a member, does not entitle them to the Register, without the payment of the usual subscription. Some may complain of this, but to avoid the possibility of running the society into debt, or commencing an enterprise which might fail for the want of funds, this course has been adopted, and after the first year, will throw all subscribers on the same footing; for after this it is hoped the subscriptions alone will sustain the work. We shall send this number to each member of the society, and hope before the issuing of the next; that we may have every member of the society as a regular subscriber. Some have given pledges to the amount of several subscriptions—we know that many might make it advantageous to themselves, and to promote the cause of Dental science, by sending it to some of their medical friends. First, however, get them if possible to subscribe, and if they will not, subscribe for them. The American Journal received ma.y subscriptions in this way. We have already enrolled some of the Medical Faculty as subscribers, and hope to enlarge the list.—CIN. ED.

### EXCHANGE LIST.

We acknowledge the receipt of several of our Western and Southern Medical periodicals, and we have no doubt we shall often find much in their pages which will be of direct interest to the Dental Surgeon. Indeed, Medicine and Dentistry are so intimately blended that it cannot be otherwise.—Cin. Ed.

